# Northampton's Feat

**Published:** 1921-02-19

**Authors:** 


### Northampton's Feat.

### OVER £30,000 QUICKLY RAISED.

We hoped to have given an account last week of the success of the Northamptonshire Hospital Week Fund, of the Hospital Workers' luncheon, and of the second annual meeting of the Northampton and County Hospital and Convalescent Fund. But the success is so astonishing that for the moment it almost took our breath away.

To begin with there was a debt of £8,379. To meet this was formed a plan of campaign, devised, we understand, by Mr. Charles H. Battle, Y.M.C.A., adopted by the Hospital Week Committee, and unstintedly supported by the Mayor and Mayoress. The town was divided into twelve wards, on the precise model of the municipal divisions, and each ward was set to raise an appropriate quotum. Carefully drawn display advertisements named the wards and the amounts of their "assessment." A "very special message" from the Mayor, Mr. W. Harvey Reeves, O.B.E., showed how the cost of maintenance had risen from £12,500 in 1914 to £30,000 in 1920. December 15 was the date on which it was hoped to free the hospital from debt. In helping to raise the Allied War Fund in Northampton, Mr. Battle had more or less won his spurs as a charity organiser.

On February 3 the second annual meeting of Hospital and Convalescent Fund took place. Being Secretary of this fund, Mr. Battle showed in his statement of accounts a balance of £6,350. On February 5 the Hospital Workers' luncheon was held at the Town Hall, by the invitation of Mr. H. Manfield, with Mr. J. H. C. Crotchett in the chair. After the loyal toasts, Mrs. Crotchett received the cheques from the county workers, which amounted to 6,000 guineas, an increase of £1,800, and the second or town fund gave £6,000, an increase of £3,500 on the sum raised last year. The joint increase, therefore, is one of £5,300. An interesting fact is that each hospital letter issued to each committee is valued at the same rate as the letters issued to annual subscribers—namely, 10s. 6d. for each out-patient letter and three guineas for each in-patient letter. All that is raised for the hospital over the equivalent of these sums is regarded as a free gift, which, for both funds, this time amounted to £9,300.

On the afternoon of February 5, at the quarterly Court of Governors, cheques amounting to £31,857 were presented to the Northampton General Hospital by the Mayor and others. The first cheque was one of £8,000 from the British Red Cross Society and the Order of St. John toward the new heating installation, and one of £3,000 toward the Margaret Spencer Home. Then came the Hospital and Convalescent Fund cheque for £6,000, and the Hospital Week Committee's cheque for £6,3C0, besides others which cannot be mentioned in detail here. The Mayor said that "their gratitude was only equal to their amazement at the enormous sums which untiring energy had raised."

